# Analyzing the Predictability of an Artificial Intelligence App (Tibot) in the Diagnosis of Dermatological Conditions: A Cross-sectional Study

**DOI:** 10.2196/45529

**Published:** 2023-03-01

**Authors:** Shiva Shankar Marri, Arun C Inamadar, Ajit B Janagond, Warood Albadri

**Affiliations:** 1 Department of Dermatology, Venereology and Leprosy Shri B M Patil Medical College, Hospital and Research Centre BLDE (Deemed to be University) Vijayapur, Karnataka India

**Keywords:** artificial intelligence, AI-assisted diagnosis, machine learning, neural network, deep learning, dermatology, mobile, application, app

## Abstract

**Background:**

Artificial intelligence (AI) aims to create programs that reproduce human cognition and processes involved in interpreting complex data. Dermatology relies on morphological features and is ideal for applying AI image recognition for assisted diagnosis. Tibot is an AI app that analyzes skin conditions and works on the principle of a convolutional neural network. Appropriate research analyzing the accuracy of such apps is necessary.

**Objective:**

This study aims to analyze the predictability of the Tibot AI app in the identification of dermatological diseases as compared to a dermatologist.

**Methods:**

This is a cross-sectional study. After taking informed consent, photographs of lesions of patients with different skin conditions were uploaded to the app. In every condition, the AI predicted three diagnoses based on probability, and these were compared with that by a dermatologist. The ability of the AI app to predict the actual diagnosis in the top one and top three anticipated diagnoses (prediction accuracy) was used to evaluate the app’s effectiveness. Sensitivity, specificity, and positive predictive value were also used to assess the app’s performance. Chi-square test was used to contrast categorical variables. *P*<.05 was considered statistically significant.

**Results:**

A total of 600 patients were included. Clinical conditions included alopecia, acne, eczema, immunological disorders, pigmentary disorders, psoriasis, infestation, tumors, and infections. In the anticipated top three diagnoses, the app’s mean prediction accuracy was 96.1% (95% CI 94.3%-97.5%), while for the exact diagnosis, it was 80.6% (95% CI 77.2%-83.7%). The prediction accuracy (top one) for alopecia, acne, pigmentary disorders, and fungal infections was 97.7%, 91.7%, 88.5%, and 82.9%, respectively. Prediction accuracy (top three) for alopecia, eczema, and tumors was 100%. The sensitivity and specificity of the app were 97% (95% CI 95%-98%) and 98% (95% CI 98%-99%), respectively. There is a statistically significant association between clinical and AI-predicted diagnoses in all conditions (*P*<.001).

**Conclusions:**

The AI app has shown promising results in diagnosing various dermatological conditions, and there is great potential for practical applicability.

## Introduction

### Background

Artificial intelligence (AI) is “the scientific understanding of the mechanisms underlying thought and intelligent behaviour, and their exemplification in machines” [1]. It aims to reproduce properties similar to human cognition [2-4]. A subset of AI where computer programs learn from experience without any definitive coding instructions is known as machine learning (ML) [1].

It may become crucial in the future for every member of the medical community to have a thorough understanding of AI, as it could be the catalyst for the transformation of health systems to increase productivity and effectiveness, providing versatility for universal health coverage, hence introducing a rudimentary change in the way we practice medicine [5,6].

The majority of diagnoses in dermatology are made primarily on visual pattern recognition and mainly depend on morphological traits. Its comprehensive clinical, dermatoscopic, and dermatopathological picture database makes it perfect for using AI image recognition skills for assisted diagnosis [1,2,7].

AI can prove to be an important tool in the screening and early diagnosis of skin cancers, thus improving the quality of care [4,8-10]. It has also been used in diagnosing and assessing various inflammatory conditions, pigmentary diseases, and hair disorders [4,9-11]. Opportunities for AI in dermatology include the potential to robotize redundant assignments, streamline tedious undertakings, improve spectator dependability issues, and eventually extend the diagnostic toolbox of dermatologists [11].

The manifestation of dermatological conditions in various forms, the lack and uneven distribution of competent dermatologists, and the need for prompt and precise diagnosis necessitate the need for an automated computer-aided diagnosis [12]. In lower-income nations like India, where there is a significant gap between the availability and demand for facilities and where the cost of health care is high, AI is especially helpful. As pandemics such as COVID-19 cannot be foreseen, it can be a noteworthy alternative for patients and doctors to use online consultations during these periods.

### About the Tibot AI App

The Tibot AI app, which is owned by Polyfins Technology Inc, was analyzed in this study. This app aims to raise awareness about skin conditions among users, categorize various skin conditions in terms of criticality, and encourage users to seek medical advice from a skin care specialist for proper treatment whenever needed. The patient’s data is safeguarded using numerous firewalls, and the users have complete control over the encrypted data. Upon sharing a photo on the app and answering a couple of questions about the skin lesion, it uses ML to break down images, inspect and compare them with similar images from its memory, and predict the most probable diagnoses.

The following are the 12 categories of skin conditions tracked by the AI app: acne and rosacea; alopecia; benign and suspicious tumors; eczema; immunological skin disorders; pigmentary disorders; psoriasis; skin infestation; and bacterial, fungal, and viral infections. The specific skin conditions tracked by the app in each category are listed in Multimedia Appendix 1. Many other apps working on a similar principle are available, and appropriate research to determine their reliability is necessary.

### Objective

This study analyzes the predictability of the Tibot AI app in the identification of dermatological diseases as compared to a dermatologist.

## Methods

### Study Design and Participant Inclusion Criteria

This observational cross-sectional study included participants of all ages consulting the dermatology outpatient department in a tertiary care facility. The skin disorders were grouped according to the categories put forward by the AI app and included acne; alopecia; benign and suspicious tumors; eczema; immunological skin disorders; pigmentary disorders; psoriasis; skin infestation; and bacterial, fungal, and viral infections. Patients with conditions warranting further evaluation, who had been treated earlier, or who were unwilling to allow their photographs to be used were excluded from this study.

### Ethics Approval

The study was conducted in accordance with the Declaration of Helsinki and approved by the Institutional Ethical Committee of BLDE (Deemed to be University; IEC/No. 09/2021 and 22/01/2021). Informed consent was obtained from all participants involved in the study.

### Methodology

A detailed history was taken, a clinical examination of the patients was performed, and a clinical diagnosis was established by an expert dermatologist. Using a smartphone camera of 12MP, images of the skin lesions were taken in confidence in a room with adequate lighting and uploaded onto the Tibot iOS app. Certain questions put forward by the app regarding the demographic profile of the patient and those pertaining to the site and duration of lesions, associated symptoms, and their intensity were answered. For every skin condition, three diagnoses were predicted by the app based on probability, and these were compared with the clinical diagnosis.

### Statistical Analysis

Percentages were used to offer a descriptive analysis of various skin problems. The efficacy of the AI app was assessed based on its potential to predict the actual diagnosis in the top one and top three anticipated diagnoses. Sensitivity, specificity, and positive predictive value (PPV) were also evaluated. Data analysis was performed using JMP Pro 16 software Version 16 (SAS Institute). Chi-square test was used to contrast categorical variables. *P*<.05 was considered statistically significant.

## Results

### Participant Characteristics

A total of 600 patients were included in the study. The majority of the patients belonged to the age group of 20-29 years (n=167, 27.8%), with a male preponderance (n=339, 56.5%). Clinical conditions included viral infections (n=111, 18.5%), alopecia (n=89, 14.8%), fungal infections (n=88, 14.7%), pigmentary disorders (n=87, 14.5%), acne and rosacea (n=73, 12.2%), psoriasis (n=47, 7.8%), benign tumors (n=28, 4.7%), eczema (n=24, 4%), immunological disorders (n=20, 3.3%), skin infestation (n=16, 2.7%), suspicious tumors (n=11, 1.8%), and bacterial infections (n=6, 1%).

### Performance of the AI App

The mean prediction accuracy for the anticipated top three diagnoses of the AI software was 96.1% (95% CI 94.3%-97.5%) and 80.6% (95% CI 77.2%-83.7%) for the precise diagnosis. The precision of diagnoses in the top three and top one predictions, known as prediction accuracy, was analyzed in each skin condition. Prediction accuracy (top one) was 97.7% for alopecia and 91.7%, 88.5%, and 82.9% for acne, pigmentary disorders, and fungal infections, respectively. Prediction accuracy (top three) was 100% for alopecia, benign tumors, eczema, and suspicious tumors (Table 1).

The AI app’s sensitivity and specificity were found to be 97% (95% CI 95%-98%) and 98% (95% CI 98%-99%), respectively.

**Figure 1:**
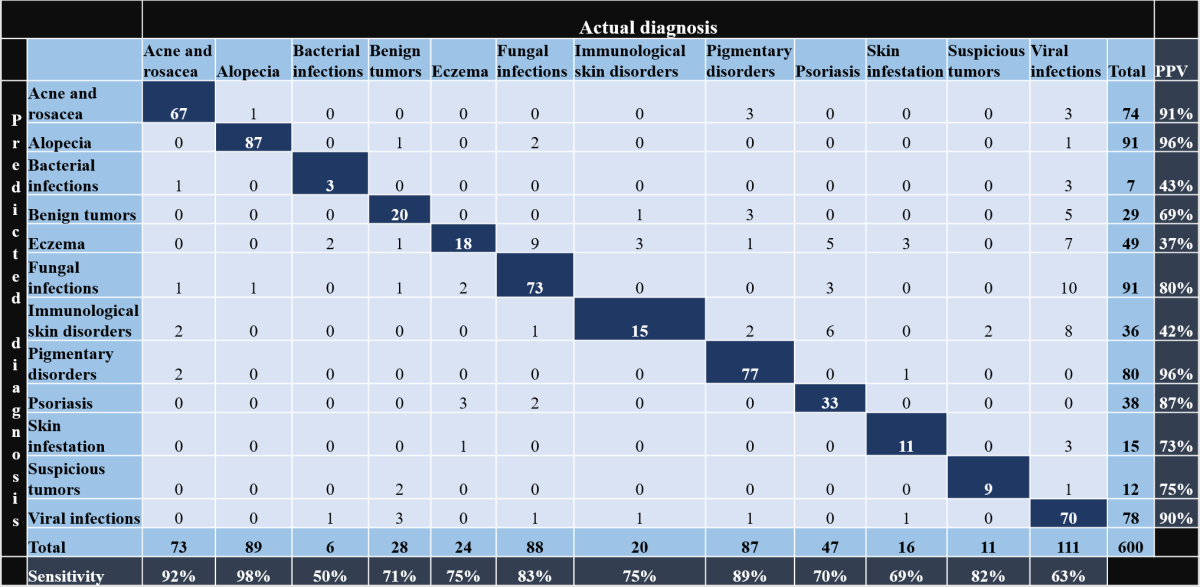
Confusion matrix of actual diagnosis versus artificial intelligence–predicted top diagnosis along with sensitivity and positive predictive value for individual skin conditions. Dark blue cells represent true positives.

**Figure 2:**
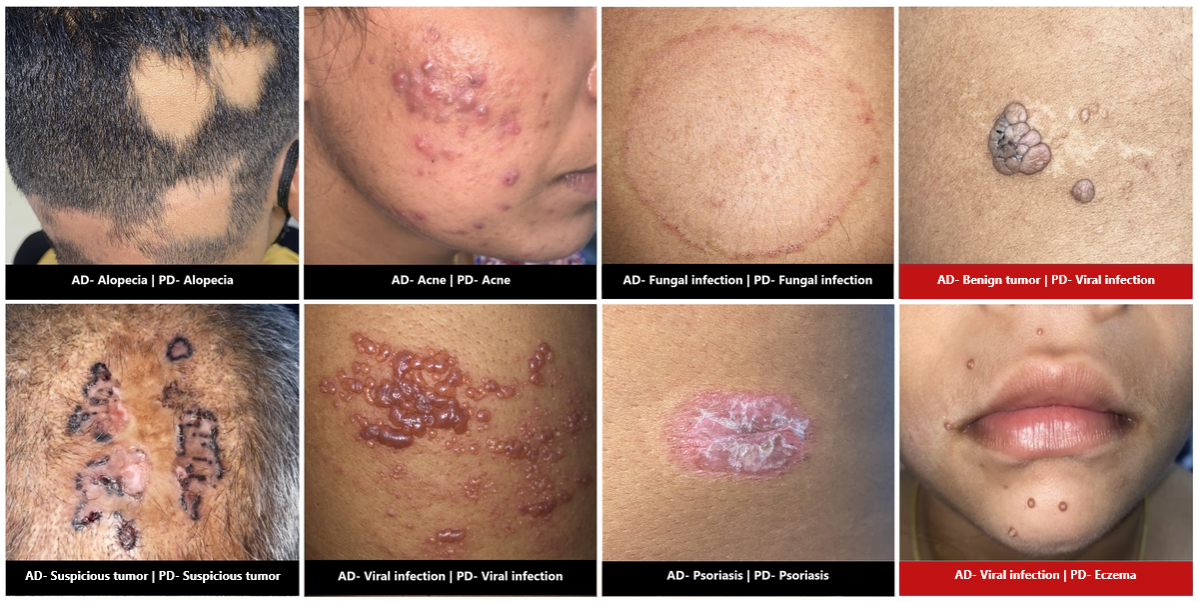
Clinical images of various skin conditions with actual and artificial intelligence–predicted diagnoses. AD: actual diagnosis; PD: predicted diagnosis.

The confusion matrix between AI-anticipated top diagnoses and actual diagnoses, along with sensitivity (top one) and PPV for individual skin conditions, is depicted in Figure 1. The sensitivity (top three) for alopecia, eczema, and benign and malignant tumors was 100%. The PPV for pigmentary disorders and alopecia was 96% and for acne and viral infections 91% and 90%, respectively. Table 2 depicts the sensitivity, PPV, and specificity of the AI app in the diagnosis of individual skin conditions. There was a statistically significant association between clinical diagnosis and predicted top diagnosis in all the conditions (*P*<.001). Figure 2 shows clinical photographs of various skin conditions with actual and predicted diagnoses.

**Table 1:** Prediction accuracy of the artificial intelligence app in top one and top three predictions in various skin conditions.

Skin conditions	Prediction accuracy (95% CI), %	
	Top one	Top three
Acne and rosacea	91.7 (82.9-96.9)	98.6 (92.6-99.9)
Alopecia	97.7 (92.1-99.7)	100 (95.9-100)
Bacterial infection	50 (11.8-88.1)	83.3 (35.9-99.6)
Benign tumor	71.4 (51.3-86.8)	100 (87.7-100)
Eczema	75 (53.3-90.2)	100 (85.8-100)
Fungal infection	82.9 (73.4-90.1)	96.5 (90.4-99.3)
Immunological skin disorder	75 (50.9-91.3)	95 (75.1-99.9)
Pigmentary disorder	88.5 (79.9-94.3)	98.8 (93.8-99.9)
Psoriasis	70.2 (55.1-82.7)	91.4 (79.6-97.6)
Skin infestation	68.7 (41.3-88.9)	93.7 (69.8-99.8)
Suspicious tumor	81.8 (48.2-97.7)	100 (71.5-100)
Viral infection	63 (53.4-72)	94.5 (88.6-97.9)

**Table 2:** The sensitivity, PPV, and specificity of artificial intelligence software for the diagnosis of various dermatoses.

Skin conditions	Sensitivity (95% CI), %			PPV^a^ (95% CI), %		Specificity (95% CI), %
	Top one	Top three				
Acne and rosacea	92 (83-97)	99 (93-99)	91 (82-95)		99 (97-99)	
Alopecia	98 (92-99)	100 (96-100)	96 (89-98)		99 (98-99)	
Bacterial infection	50 (12-88)	83 (36-99)	43 (17-77)		99 (98-99)	
Benign tumor	71 (51-87)	100 (88-100)	69 (53-82)		98 (97-99)	
Eczema	75 (53-90)	100 (86-100)	37 (28-47)		95 (92-96)	
Fungal infection	83 (73-90)	97 (90-99)	80 (72-87)		96 (94-98)	
Immunological skin disorder	75 (51-91)	95 (75-99)	42 (30-54)		96 (95-98)	
Pigmentary disorder	89 (80-94)	99 (94-99)	96 (89-99)		99 (98-99)	
Psoriasis	70 (55-83)	91 (80-98)	87 (73-94)		99 (98-99)	
Skin infestation	69 (41-89)	94 (70-99)	75 (50-89)		99 (98-99)	
Suspicious tumor	82 (48-98)	100 (72-100)	75 (48-91)		99 (98-99)	
Viral infection	63 (53-72)	95 (89-98)	90 (81-95)		98 (97-99)	

^a^PPV: positive predictive value.

## Discussion

### Principal Findings

In this study, we have analyzed the diagnostic precision of the Tibot AI app for a variety of dermatological disorders as compared to that of a dermatologist. The app works on the principle of a convolutional neural network (CNN), which breaks down images into numerical forms and compares them with similar images from its own memory to predict the probable diagnosis [4,8].

This study included the maximum number of patients with infections, alopecia, and pigmentary disorders. The app’s mean prediction accuracy was 96.1% (95% CI 94.3%-97.5%) for identifying and predicting the diagnosis in the top three predictions and 80.6% (95% CI 77.2%-83.7%) for the exact diagnosis. The prediction accuracy (top 1) was 97.7%, 91.7%, 88.5%, and 82.9% for alopecia, acne, pigmentary disorders, and fungal infections, respectively. The prediction accuracy (top 3) was 100% for alopecia, eczema, and benign and suspicious tumors. The PPV was 96% for alopecia and pigmentary disorders and 91%, 90%, and 87% for acne, viral infections, and psoriasis, respectively.

### Comparison With Previous Studies

A study done by Patil et al [4] assessed the Tibot AI app in 398 patients. The study showed that the mean prediction accuracy of the app was 60.7% for the precise diagnosis and 85.2% to predict the actual diagnosis in the anticipated top three. A better mean prediction accuracy of 96.1% and 80.6% was demonstrated in our study in the top three and top one predictions, respectively. The comparison of the prediction accuracy (top three) and PPV of this study and their study is depicted in Table 3. The prediction accuracy in individual skin conditions was also significantly better in our study. The PPV was comparable in most of the conditions. The sensitivity of the AI app was 86% in their study as compared to 97% in our study, whereas the specificity was 98% in both studies. Suspicious tumors were not included in their study.

AI has an active learning process that allows the app to expand the database, ultimately improving the predictive accuracy and diagnostic ability [4]. The better results in our study could be attributed to this nature, along with software updates of the app and variations in the quality of the pictures uploaded. By submitting clinical images and responding to a few key questions pertaining to the location, duration, and intensity of the associated symptoms of the lesions, these dermatoses are evaluated. The different visual manifestations of the same disorder among various patients and the subjective nature of symptoms can be attributed to the discrepancy in accuracy.

A study conducted by Wu et al [13] assessed a CNN model in diagnosing inflammatory skin conditions such as psoriasis, atopic dermatitis, and eczema. The model was trained based on 4740 clinical images. The overall accuracy of the application was 95.8%, with a sensitivity of 94.4% and a specificity of 97.2%. The accuracy for psoriasis was 89.46% and for atopic dermatitis and eczema 92.57%. In our study, the app showed a prediction accuracy (top one) of 75% and 70.2% for eczema and psoriasis, respectively.

Fujisawa et al [14] assessed a CNN that was trained using a data set of 4867 images in classifying skin tumors into benign and malignant as compared to board-certified dermatologists. The overall classification accuracy was 76.5%, with a sensitivity of 96.3% and a specificity of 89.5%.

A study on the classification of skin cancer using deep neural networks was published by Esteva et al [15]. They trained a CNN using 129,450 clinical pictures comprising 2032 distinct illnesses. This app diagnosed melanomas and keratinocyte carcinomas with an overall accuracy of 72.1%, with a comparable or better efficacy than 21 dermatologists.

Han et al [16] developed an automated classification system for 12 established benign and malignant dermatoses using 19,398 images. The CNN performed comparably to 16 dermatologists. For all conditions, the average sensitivity and specificity were 85.1% and 81.3%, respectively.

Our study included 39 skin tumors, of which 11 were suspicious and 28 were benign. For suspicious tumors, the prediction accuracy (top one) and PPV were 81.8% and 75%, respectively, while for benign tumors, it was found to be 71.4% and 69%, respectively. The prediction accuracy (top three) and sensitivity for both were 100%. Although these conditions ideally require histopathological confirmation, the tumors included in this study were clinically apparent.

**Table 3:** Comparison of the prediction accuracy and PPV of artificial intelligence software in this study and the study by Patil et al [4].

	Current study, %		Study by Patil et al [4], %	
	Prediction accuracy	PPV^a^	Prediction accuracy	PPV
Acne and rosacea	98.6	91	84	87.5
Alopecia	100	96	100	100
Bacterial infection	83.3	43	78.9	78.9
Benign tumor	100	69	71.4	83.3
Eczema	100	37	91.7	94.3
Fungal infection	96.5	80	95.6	90
Immunological skin disorder	95	42	88.9	42.1
Pigmentary disorders	98.8	96	75	75
Psoriasis	91.4	87	73.7	82.3
Skin infestation	93.7	73	63	94.4
Suspicious tumor	100	75	—^b^	—
Viral infection	94.5	90	26.7	80

^a^PPV: positive predictive value.

^b^Suspicious tumors were not included in their study.

### Nomenclature and Inception of AI

AI is a broad scientific discipline dedicated to developing programs that display properties of human intellect and encompasses ML and deep learning [3,6,17,18]. ML includes various approaches that enable algorithms to learn from data without explicit programming [1,10,17]. A supervised, semisupervised, or unsupervised learning process can be used. A subtype of ML called deep learning incorporates artificial neural networks that mimic the structure of biological neural networks in the brain [1,8].

Artificial neural networks or neural networks are flexible mathematical models that identify complex nonlinear relationships in large data sets. They are arranged in three layers—input, hidden, and output. A “deep” neural network has three or more concealed layers. The input layer receives relevant data, which is then processed through multiple layers of hidden algorithmic processes, where each layer detects some feature within the input [1,5,8,11]. A special category of artificial neural networks known as CNNs includes three types of layers—convolution, pooling, and fully connected [8,18].

Since the dawn of time, man has relied on machines to help him live and simplify his existence. Inferential statistics are used in the medical field to confirm or refute hypotheses that have been developed via observation and analysis of the patients. AI expands on this strategy by recognizing patterns that are difficult for humans to notice [5]. Although AI in dermatology was focused on pigmented skin lesions and melanoma detection initially, newer algorithms have since been developed with a wide range of applications, including collecting images, decoding, evaluation, report generation, and strategizing for follow-up [11,17].

### Limitations

The limitations of our study include a lack of standardization of images with respect to focus, angle, and lighting, and the inability to record skin type. Assessment of various Fitzpatrick skin types is required to corroborate the findings. Regarding the composition of study participants, the majority had infections, alopecia, pigmentary disorders, and acne. Although AI recognition of skin tumors in this study is feasible, further studies with more participants are required, as a small sample size has less validity.

Currently, AI in dermatology, including the Tibot app, can only recognize a group of explicit skin diseases with a lack of a specific diagnosis [2,11]. To construct the ideal app, close collaboration among multidisciplinary experts in the domains of computer science, biomedicine, and medicine is essential [2,19]. There are unresolved legal, ethical, privacy, and liability issues associated with AI diagnosis that might hinder regulatory approval [2,3,11,18,20]. Another hurdle that AI faces is public trust and acceptance as people struggle to understand the decision-making process of AI, referred to as a black box [11,18,20]. A particular issue that is understudied in the existing research on the principles of using AI in health services is the absence of explicit and unique descriptions of the notions used to construct the algorithms, as well as how their core concept may be translated into machine code and subsequently interpreted by doctors [21]. Dermatology, being primarily a visual specialty, is also a tactile one [2]. Therefore, predicting the diagnosis based solely on 2D photographs is difficult. These are some of the drawbacks of AI in dermatology.

### The AI Conundrum

This new era of AI-augmented practice has its fair share of skeptics and supporters. While AI might seem threatening to dermatologists’ diagnostic skill set, it is important to remember that AI can only provide a probability of broad diagnoses and certainly not the treatment nor can it provide humanistic care. To achieve the utmost potential of AI, developers must strive to create algorithms that represent a variety of patient populations, ensure that the output is ultimately comprehensible, prospectively validate its performance, provide doctor-patient interaction when necessary, and demonstrate authenticity to the regulatory bodies.

### Conclusion

The Tibot AI app has shown encouraging results in diagnosing various dermatological conditions. There is great potential for practical applicability, although further improvement is required for its implementation in clinical practice. Its greatest strength is the ability to learn independently without human intervention.

AI is by no means an equivalent to the correspondence between doctors and patients neither can it offer medical care or human touch, but it is an important aid to dermatologists and patients. The public’s lack of inclination to adopt a contentious technology will be the biggest barrier to its general acceptance. While it is helpful in broadly categorizing diseases, detailed knowledge of the subject and its implementation in the correlation of various diseases will still be needed for fine-grained diagnosis and further management. Rather than succumbing to the fear of a dystopian future where AI replaces dermatologists, it is imperative that we should embrace it and incorporate it into patient care paradigms.

## Appendix

Multimedia Appendix 1 Specific skin conditions tracked by the artificial intelligence application in each clinical category.

## Abbreviations

AI: artificial intelligence
CNN: convolutional neural network
ML: machine learning
PPV: positive predictive value
