# Supplementary material for: Analyzing the Predictability of an Artificial Intelligence App (Tibot) in the Diagnosis of Dermatological Conditions: A Cross-sectional Study
Source: JMIR Dermatol. 2023 Mar 1;6:e45529. doi: 10.2196/45529 (PMC10335135; doi:10.2196/45529)
Supplement: Multimedia Appendix 1 [file derma_v6i1e45529_app1.docx]

Multimedia Appendix 1: Specific skin conditions tracked by the AI application in each clinical category

| S. No.: | Clinical categories | Specific skin conditions tracked by the application |
| --- | --- | --- |
| 1. | Acne & Rosacea | Acne vulgaris  Acne conglobata  Acne excoriee  Neonatal acne  Perioral dermatitis  Rosacea |
| 2. | Alopecia | Pattern alopecia (MPHL^a^, FPHL^b^)  Alopecia areata  Anagen effluvium  Traction alopecia  Telogen effluvium  Scarring alopecia |
| 3. | Bacterial infection | Folliculitis  Impetigo  Ecthyma  Carbuncle  Cellulitis  Erysipelas  Erythrasma  Leprosy  STD^c^ |
| 4. | Benign tumors | Seborrheic warts  Melanocytic nevi  Vascular malformations  Dermatofibroma  Neural tumours  Lipoma  Mastocytosis  Cysts  Mucocele  Keloid  Xanthelasma  Acrochordon  Dermatosis Papulosa Nigra |
| 5. | Eczema | Asteatotic eczema  Atopic dermatitis  Contact dermatitis  Lichen simplex  Nummular eczema  Pityriasis alba  Pompholyx  Seborrheic dermatitis  Venous eczema |
| 6. | Fungal infections | Candidiasis  Tinea  Cryptococcosis  Mycetoma  Oychomycosis  Pityriasis versicolor |
| 7. | Immunological skin disorders | Bullous pemphigoid  Pemphigus  Dermatitis herpetiformis  Dermatomyositis  Drug eruptions  Lichen planus  Lupus erythematosus  Morphea  Systemic sclerosis  Urticaria |
| 8. | Pigmentary disorders | Acanthosis nigricans  Albinism  Freckles  Melasma  Periorbital hypermelanosis  Vitiligo |
| 9. | Psoriasis | Plaque psoriasis  Pustular psoriasis  Guttate psoriasis  Inverse psoriasis |
| 10. | Skin infestations | Insect bites  Leishmaniasis  Cutaneous myiasis  Onchocerciasis  Pediculosis  Scabies |
| 11. | Suspicious tumors | Basal cell carcinoma  Keratoacanthoma  Melanoma  Solar keratoses  Squamous cell carcinoma |
| 12. | Viral infections | Herpes simplex 1 & 2  Molluscum contagiosum  Shingles  Viral exanthem  Warts |

^a^MPHL: Male pattern hair loss

^b^FPHL: Female pattern hair loss

^c^STD: Sexually transmitted diseases
